# The Impact of Short‐Term Leadership Study Abroad on Student Leadership Competencies

**DOI:** 10.1002/yd.70037

**Published:** 2026-02-17

**Authors:** Jessica W. Hench, Eden D. Baroody

**Affiliations:** ^1^ Department of Leadership and American Studies Christopher Newport University Newport News Virginia USA; ^2^ Department of Student Engagement Germanna Community College Fredericksburg Virginia USA

## Abstract

Short‐term study abroad programs have become increasingly popular in US higher education, as they offer powerful development opportunities for undergraduate students. This mixed methods study examined the impact of three faculty‐led short‐term summer study abroad courses in a leadership studies curriculum. Through the lens of Seemiller's student leadership competencies, survey data from six student cohorts revealed growth in key competency clusters, particularly *personal behavior*, *self‐awareness and development*, *civic responsibility*, and *learning and reasoning*. Short‐term study abroad helped students develop leadership skills such as functioning independently, responding to ambiguity, emotional intelligence, and social responsibility. These leadership competencies help augment students’ college experiences, as well as provide them with enhanced career readiness. These findings support the integration of study abroad into leadership development curricula and suggest the use of competency‐based frameworks for intentional program design.

## 1 Introduction

For traditional‐age students, higher education marks a pivotal time ripe with opportunities for professional and personal growth. “The college experience is a transformational time between adolescence and adulthood, a time when young people develop their full potential and make major decisions about their life's course” (Hench 2015, 12). A major aspect of contemporary higher education is leadership development, which is increasingly important for employability and career success (NACE 2026). Thus, increasing numbers of colleges and universities are incorporating intentional leadership development programs into their offerings through both academic and cocurricular experiences (Guthrie et al. 2018). Leadership studies are a natural fit for a college education, as there exists a “…symbiotic relationship between liberal education and leadership studies” (Colvin 2003, 34).

Another component of higher education that has long been known to benefit students is study abroad, which continues to grow in popularity (Taylor & Teig 2026). Beyond traditional semester‐long study abroad experiences, many institutions are offering short‐term study abroad programs, which appeal to a broader range of students (Mapp 2012). In 2024, 60% of students who studied abroad completed short‐term programs of eight weeks or less during the summer or academic year (Institute of International Education 2024).

The research presented here explored the combination of an undergraduate leadership program and study abroad experience. This study is framed by Seemiller's (2014) student leadership competencies (SLCs), defined as “…knowledge, values, abilities, and behaviors that help an individual contribute to or successfully engage in a role or task” (p. xv). This mixed‐methods study explored the impact of faculty‐led short‐term leadership study abroad courses on the leadership development of undergraduate students.

## 2 Study Abroad and Leadership Learning Literature

As colleges and universities continue to emphasize global learning and student development, study abroad has become increasingly popular. Short‐term study abroad experiences have emerged as a particularly accessible and impactful format for undergraduate students. This literature review examines existing scholarship on short‐term study abroad programs, with particular attention to their role in fostering leadership development, cultural competence, and career readiness. These outcomes align with the goals of leadership education and provide a foundation for understanding the potential impact of faculty‐led study abroad within a leadership studies academic curriculum.

### 2.1 Short‐Term Study Abroad Experiences

The number of undergraduate students studying abroad during their college degree program has steadily increased over the years, even approaching pre‐pandemic enrollment (Institute of International Education 2024). According to the *2024 Open Doors Report* (Institute of International Education 2024), in the 2022–2023 academic year, 280,716 US students studied abroad for academic credit; as a whole, nearly 10% of all US undergraduate students studied abroad during their degree program. Over 60% of these students completed short‐term study abroad programs of eight weeks or less during the summer or academic year (Institute of International Education 2024), making short‐term programs particularly worthy of attention.

Short‐term study‐abroad programs can often be more appealing to students than semester or year‐long programs, as they may be more financially feasible, more accessible to student‐athletes and others who cannot be away from campus for a whole semester or year, and more favorable for inexperienced travelers who do not yet have the confidence to travel alone or for longer durations (Coste and Witt 2026; Mapp 2012). In shorter courses, faculty have more control over student activities and can better gauge how to plan the itinerary, allowing for more purposeful learning to occur (Mapp 2012; Teig 2026).

### 2.2 Impact on Leadership and Personal Development

In addition to academic learning, study abroad experiences benefit students in their leadership development. The Council for the Advancement of Standards in Higher Education (CAS) stated the goals of leadership programs in colleges and universities should include foundations of leadership, personal development, interpersonal development, and the development of groups, organizations, and systems (CAS 2012). Studying abroad can help augment these goals.

A qualitative study of a four‐week study abroad experience found that undergraduate students grew in their leadership identity through student‐faculty interactions, critical thinking, connecting course content to real‐life experiences, learning about a new culture, and expanding their comfort zones (Beatty and Manning‐Ouellette 2022). Having a well‐developed leadership identity is an essential element of engaging in the leadership process. This includes leadership capacity, which is the integration of students’ knowledge, skills, and attitudes into the leadership process (Dugan et al. 2011).

Leadership identity also includes leadership self‐efficacy (LSE) which is a student's belief that they are capable of succeeding in leadership (Dugan et al. 2013). From a psychological perspective, study abroad experience leads to gains in the intrapersonal, interpersonal, and cognitive domains of human development (Redwine et al. 2018), all of which support the cultivation of leadership identity. Additionally, Manning‐Oullette (2024) explained how college leadership programs can provide experiences for training in cultural contexts, stating, “…study abroad laboratories offer hands‐on experiences that can center the cultivation of leadership skills” (p. 44). When done correctly, this leads to cultural appreciation.

### 2.3 Cultural Appreciation

In a phenomenological case study of a four‐year undergraduate leadership program, Hench (2015) found study abroad to be an impactful experience for participants, allowing them to see the world more broadly, interact with diverse others, experience new cultures, and develop an interest in learning about people different from themselves. Other findings similarly purport to show the cultivation of leadership development, as study abroad increases intercultural knowledge and competence (Beatty and Manning‐Ouellette 2022). These skills are becoming more important as the world becomes increasingly globalized.

Study abroad experiences provide an opportunity for leadership students to “…delve into a unique, powerful cultural experience and share a deep learning about leadership through immersion and reflection” (Montgomery and Arendorf 2012, 67). A resident of any particular country is limited to how the world looks from their point of view; in the case of this study, a Western and specifically American viewpoint. Visiting a new country for an extended amount of time pushes people to test their beliefs and assumptions of the world (Desmond 2014). Noda (2007) describes the significant impact of “performed culture” (p. 298) study abroad provides; these are the experiences of everyday life such as navigating shops, restaurants, transportation, cultural roles and norms, and casual social interactions. Students are pushed to perform daily tasks in a new setting that may be similar or drastically different from their own lives.

### 2.4 Employability and Long‐Term Impacts

Even short‐term study abroad experiences may have valuable long‐term impact. To be career‐ready in an increasingly globalized world, college students need to take advantage of everything their education can provide (LeGrant 2017). Many employers value study abroad experience, as it helps students develop career competencies like communication, teamwork, and professionalism, and students pursuing postgraduate education (Toner 2019). The National Association of Colleges and Employers (NACE) defined career readiness as, “…a foundation from which to demonstrate requisite core competencies that broadly prepare the college educated for success in the workplace and lifelong career management” (NACE 2026).

NACE (2026) also outlined eight career readiness competencies that have been identified as essential to workplace success and that can be developed through the higher education experience: career and self‐development, leadership, communication, critical thinking, equity and inclusion, professionalism, teamwork, and technology. The benefits of studying abroad resonate strongly with the career readiness competencies and the goals of student leadership development programs.

## 3 Conceptual Framework

While leadership development is an essential component of higher education, it is hard to quantify. To help frame the growth and development of undergraduate students, Seemiller (2014) developed 60 SLCs, defined as “…knowledge, values, abilities, and behaviors that help an individual contribute to or successfully engage in a role or task” (p. xv). Competencies have been used in corporate and professional settings for years and have since moved into the higher education context (Seemiller 2016).

Seemiller's (2014) competencies are based on extensive research of a variety of competency models and student leadership programs throughout the United States. Croft and Seemiller (2017) aptly stated, “colleges and universities provide an ideal environment for helping students develop leadership competencies before they enter their future careers” (p. 10). Within the SLC framework, the 60 competencies are grouped into eight clusters: *learning and reasoning, self‐awareness and development, interpersonal interaction, group dynamics, civic responsibility, communication, strategic planning*, and *personal behavior* (Croft and Seemiller 2017).

To further support the use of these competencies, Seemiller (2014) developed *The Student Leadership Competencies Guidebook* to be used as a tool for developing a program or course. Croft and Seemiller (2017) also provided examples of the SLCs being used in specific programs in US colleges and universities, including both cocurricular and academic leadership programs. The benefits of using these competencies include providing a theoretical foundation for building programs and experiences for leadership education, giving students the language to communicate their development with future employers, and creating a systematic way to collect data for measuring an otherwise elusive construct (Croft and Seemiller 2017).

## 4 Research Design and Methodology

The purpose of this study was to analyze the impact of short‐term study abroad programs in leadership studies based on Seemiller's SLCs. The researchers hypothesized study abroad would enhance students’ development, and the study aimed to determine which of the SLCs were most impacted and how. This mixed methods case study design used both quantitative and qualitative elements, focusing primarily on qualitative analysis to explore students’ lived experiences.

## 5 The Leadership Program

This study focused on a robust undergraduate leadership program at a regional public university. The four‐year program provides a merit‐based scholarship with a minimum GPA requirement. At the time of this study, leadership students made up nearly 30% of the university's student body. The cocurricular component of the program consists of experiential learning, personal development, and community engagement. For the academic component of the program, students complete an 18‐credit minor in Leadership Studies, with the option to pursue a 30‐credit second major. The academic curriculum includes a set of core courses grounded in leadership theory and practice, as well as a range of elective offerings with an interdisciplinary approach to prepare students to effectively engage in the leadership process within any career field.

As outlined in the *2024–2025 Co‐Curricular Handbook* provided by program staff, the leadership program supports the mission of the university to engage students in meaningful real‐world experiences to prepare them to become engaged citizens and leaders. Each of the cocurricular activities in the four‐year program is aligned with one or more of Seemiller's SLCs, thus making this model an ideal conceptual framework for this study.

### 5.1 Short‐Term Study Abroad Courses in Leadership Studies

In addition to the on‐campus program components, leadership students are encouraged to study abroad and are eligible for financial support to help defray costs. In the 2023–2024 academic year, the university sent 268 students to over 33 countries, 133 of whom were leadership students. The leadership studies academic program offers several faculty‐led, credit‐bearing courses focused on a variety of leadership topics in diverse regions of the world. This study focused on three short‐term faculty‐led, three‐credit study abroad courses in leadership studies that took place in the summers of 2022 and 2023.

One course was a three‐week study in the Czech Republic, where students were immersed in the lives and influence of key leaders throughout Czech history. Visiting historic sites related to the leaders studied provided rich context to the history beyond the classroom. Next, a 15‐day course taught students about social justice in South Africa, with a focus on learning and reflecting on the country's culture. Lastly, a three‐week course in the Netherlands and neighboring Western European countries was designed for students to explore the concepts of globalization, international organizations, and the influences of cultural values. Each course was led by two faculty from the home university's Department of Leadership Studies, as well as some colleagues from other disciplines. Student participants represented a variety of majors from the humanities to the sciences, with the common denominator being a major or minor in leadership studies.

### 5.2 Sampling

While the university offers numerous study abroad courses, this study was limited to courses specifically granting credit in leadership studies. The sample consisted of students who participated in these three specific short‐term faculty‐led, three‐credit leadership studies courses that took place in the summers of 2022 and 2023. This made a total of six program cohorts over two years, for a total of 104 students. The researchers asked program faculty to send a survey via email to their program cohorts for two reasons; first, so the researchers would not be collecting identifying information from those students, and second, as students would be more likely to respond to faculty with whom they studied abroad. A total of 37 students, approximately 35%, responded to the survey, providing rich data for analysis.

### 5.3 Survey Instrument

The research team developed a Qualtrics survey to distribute via email to the participants. The survey included 25 Likert‐type competency statements aligned with Seemiller's (2014) SLCs. Participants were given the directions:

*Reflect on your study abroad experience thoughtfully and consider how it impacted your development in the following areas. Use the following statement to respond to each item: “My study abroad experience increased my ability to _________.”*

Items included such statements as “consider perspectives other than my own” and “engage in self‐development experiences.” For each statement, participants were prompted to select *disagree, unsure, somewhat agree*, or *strongly agree. *The final section of the survey consisted of two open‐ended short answer questions designed to provide qualitative personal examples. The questions were: (1) *How have you changed as a result of this experience?* and (2) *What was most meaningful about your study abroad experience?* These questions were intentionally broad to elicit authentic responses from students.

### 5.4 Data Analysis

The Qualtrics survey data was analyzed in two parts. First, the 25 competency statement responses were reviewed and quantified, sorting them by most frequent responses of ‘strongly agree’ and ‘agree’ to determine which SLC competency clusters were most strongly represented. The written responses to the two open‐ended short‐answer questions were reviewed qualitatively using directed content analysis (Hsieh and Shannon 2005). Responses were coded for key words and concepts related to the 60 specific SLCs (Seemiller 2014), then sorted thematically by competency cluster.

## 6 Findings

Analysis of the 25 competency statements revealed that certain competency clusters stood out most distinctly. The sampled students’ short‐term leadership study abroad experiences most strongly impacted them in terms of the SLC clusters *personal behavior* (20.4%)*, self‐awareness and development* (15.1%), *civic responsibility* (13.8%), and *learning and reasoning* (13.2%). This quantitative data was supported and enhanced by the short‐answer responses, which provided valuable qualitative feedback, as students wrote in their own words about their lived experiences. Interestingly, even without specific prompting, students mentioned examples of the SLCs in their responses.

### 6.1 Personal Behavior

Findings showed approximately 20% of respondents indicated their abilities increased in the competency cluster of *personal behavior* as a result of their study abroad experience. Specifically, they indicated growth in the competencies of *functioning independently*, *responding to ambiguity*, and *confidence*.

The *functioning independently* competency is defined as “…being able to function without assistance or guidance from others, such as finding answers on one's own” (Seemiller 2014, 120). One student wrote, “Studying abroad gave me a new sense of confidence and independence! Navigating a new city with a new group of people was challenging at times but led to so much growth in myself.” Students also needed to manage their time in order to engage in course activities as well as their social experiences and independent travel during free time. Another student wrote:

I loved the time we had away from the classroom where we could go into the city without a chaperone and truly figure out how to navigate ourselves in a different city with a different culture. I was able to learn so much from interacting with locals in these situations as I heard all of their interesting stories and lives.

These experiences reflect the impact of “performed culture” described by Noda (2007), as students navigated transportation, shopped in stores and local markets, dined in restaurants, and observed cultural norms.

Another competency that emerged in the findings was *responding to ambiguity*, “being able to effectively respond to an unpredictable situation, including adapting one's plans at the last minute, shifting gears as new information is presented, and/or moving forward without all the information” (Seemiller 2014, 129). Complementing this competency is *resilience*, which includes rebounding from setbacks and becoming “better able to face stress, challenges, and adversity in the future” (Seemiller 2014, 133). These competencies were strengthened as study abroad pushed students into situations where they could not predict or control everything happening around them. One student reflected, “Since studying abroad, I have been able to step out of my comfort zone more often because it showed me that there is so much to be gained from experiences that may seem daunting or scary at first.” This suggests that even short‐term study abroad can have a lasting impact on students’ growth beyond the course experience.

Many participants strongly agreed that study abroad increased their *confidence*, “being able to appear certain of one's beliefs, knowledge, convictions, and/or capabilities” (Seemiller 2014, 137). One student wrote, “I feel like I have become more confident in who I am as a person. I am also more happy with who I am.” Another reflected, “This experience has helped me to see the benefit of putting myself outside of my comfort zone for growth.” Anecdotally, the researchers and faculty have observed how students who participated in short‐term study abroad were likely to complete additional short‐term study abroad programs, explore opportunities to travel to academic conferences and other scholarly events, pursue their own travel experiences, and even garner the courage to spend a full semester abroad.

These experiences related to the *personal behavior* competency reflect the aims of higher education to increase personal and interpersonal development (CAS 2012), build LSE (Dugan et al. 2013), and expand the intrapersonal, interpersonal, and cognitive domains of human development (Redwine et al. 2018).

### 6.2 Self‐Awareness and Development

Similarly to personal behavior, findings also showed how students recognized their growth in the competency cluster of s*elf‐awareness and development*, comprising the second highest percentage (15.1%) of responses to the competency statements.

Specific competencies in this cluster included *self‐development*, defined as “being motivated to engage in self‐development opportunities to achieve one's fullest potential and benefit oneself and others” (Seemiller 2014, 35). One student wrote, “This may sound odd, but I feel like being in a new country almost helped me solidify who I am as a person.” This reflects *self‐development* as well as the competency of *self‐understanding*, “being motivated to enhance one's understanding of one's personality, beliefs, capacities, and interests so as to develop a greater depth of understanding of oneself in order to engage in more authentic and productive behavior” (Seemiller 2014, 24). Students’ awareness of their development in these competencies supports research on increased leadership identity and self‐efficacy (Dugan et al. 2011; Dugan et al. 2013), as well as intrapersonal development (Redwine et al. 2018) as key features of leadership development supported by the study abroad experience.

Student responses also reflected the *personal values* competency, “understanding that acting in alignment with one's own values can contribute to one's authenticity as well as inspire others” (Seemiller 2014, 26). This competency was specifically addressed in the open‐ended short answer responses. A student wrote, “We talked a lot about values and morals in our study abroad classes, and I think my value system deepened as a result of studying abroad,” and another reflected, “It strengthened my values and caused me to contemplate those of others.” Findings related to the s*elf‐awareness and development* competency cluster reflected findings from past studies, revealing that short‐term study abroad increased leadership identity through connecting course content to real‐life experiences, learning about a new culture, and expanding their comfort zones (Beatty and Manning‐Ouellette 2022).

### 6.3 Civic Responsibility

Approximately 14% of students demonstrated gains reflecting the cluster of *c* *ivic responsibility*, specifically recognizing growth in three specific competencies. *Diversity* entails, “Understanding the importance of having exposure to people from different backgrounds, beliefs, and/or experiences” (Seemiller 2014, 74). A student reflected profoundly, “I think the most meaningful part of the experience for me was when I realized just how naive I was to certain events that had happened in other parts of the world. For me, this was a really hard thing to face because I realized my privilege and that can be a hard thing to come to terms with. I can remember the exact moment when the realization hit me, and I will forever remember that feeling.”

Coupled with diversity, *inclusion* involves believing in the importance of and engaging in “ways to cultivate a welcoming environment that includes others in roles, processes, and experiences to foster a greater sense of belonging and/or shared commitment” (Seemiller 2014, 78). This growth was reflected in many student responses, like this one: “This experience revealed to me the impact my culture has on my life. It exposed me to different ideas and lifestyles.” Another wrote:

We hear so much in the news about the world at large, and we are so easily connected with those abroad via the internet. But actually going abroad and experiencing what the culture is like, how they live, how their governments operate, is so amazing. I've realized just how different yet similar the world at large actually is.

Additionally, study abroad enables growth in the competency of *social responsibility*. Seemiller (2014) explained, “…it is vital that leaders engage in responsible decision making and ethical actions so that the impact they leave benefits and does not detract from the welfare of society and its members” (p. 81). One student reflected, “I think being able to meet so many people from so many different backgrounds was so important to understand the different cultures and reality of life in different countries.” These findings support the impact of study abroad on helping students see the world more broadly as well as interacting with and developing an interest in learning about diverse others (Hench 2015). It also reflects growth in intercultural knowledge and competence (Beatty and Manning‐Ouellette 2022) and testing one's beliefs and assumptions (Desmond 2014).

Growth in the *c* *ivic responsibility* cluster reflects the NACE (2026) career readiness competencies of equity and inclusion, the “awareness, attitude, knowledge, and skills required to equitably engage and include people from different cultures and backgrounds,” which are highly valued in a globalized workforce. In a particularly noteworthy response, a student wrote, “In a noneducational sense, I absolutely loved being exposed to a new culture and people and learning to adapt and live in the way the people there do. Becoming more of a national citizen in a country that is not my own or my home.” It is interesting that this student did not see this portion of their learning as ‘educational,’ yet the natural byproduct of study abroad was tremendous personal learning, exactly as leadership educators should hope to achieve.

### 6.4 Learning and Reasoning

The competency cluster of *learning and reasoning* was supported by about 13% of the responses. One student reflected on the most meaningful part of their experience abroad:

The chance to meet and engage with others of vastly different cultures, histories, and values proved invaluable in helping me to discover my own place in the world. It represents the opportunity to test your own values (conscious and subconscious) against a much larger sample size than you could in America.

This directly reflects the competency of *other perspectives*, “considering perspectives other than one's own and allowing new information, differing opinions, and others’ experiences to impress upon one's thinking and understanding and appreciation of others” (Seemiller 2014, 4).

While the students’ responses seemed to reflect most highly their personal growth, it is also vital to consider that learning about leadership beyond the traditional classroom enables students to interact with course content in new ways. One student wrote, “the class content itself was so much more impactful to me on this trip than it would have been in a classroom because of how immersed we were in it.” Another reflected:

Learning the history first‐hand, seeing where it took place, and even the opportunity to speak to people who witnessed it directly. The whole experience was extremely rewarding and eye opening. The information I studied has stuck with me easily and I was genuinely excited to learn more each day.

Short‐term leadership study abroad courses offer unique opportunities for developing the SLC of *learning and reasoning*, which also supports the NACE (2026) career readiness competencies of career and self‐development, leadership, communication, and critical thinking.

### 6.5 Group Dynamics and Interpersonal Interaction

While the competency statements elicited relatively fewer responses related to the impacts of *g* *roup dynamics* (11%) and *interpersonal interaction* (9.8%), these competency clusters are heavily reflected in the qualitative short‐answer responses.

When asked what was most meaningful about their study abroad experience, many students wrote about interactions with their peers. For example, one said, “Being with my friends in a new place with two wonderful professors and learning about different cultures was incredible, and we all formed amazing relationships.” Many students wrote about building friendships, continuing their connections back on campus, and meaningful interactions with faculty. This supports prior research findings that “student‐faculty interactions contributed significantly to building leader capacity” (Beatty and Manning‐Ouellette 2022, 7).

The *g* *roup dynamics* and *interpersonal interaction* competency areas support the CAS (2012) standards for college leadership programs to include interpersonal development and the development of groups, organizations, and systems, as well as the NACE (2026) career readiness competencies of teamwork and equity and inclusion. One student wrote poignantly, “I made connections and memories that I'll treasure forever, as well as learned lessons that changed my views on the greater world.”

### 6.6 Other Competencies

The competency clusters of *s* *trategic planning* and *communication* were mentioned less explicitly by students in this sample, yet there is evidence they were still impacted in small ways. It can be presumed that only 7.2% of students mentioning *s* *trategic planning* is likely due to the faculty‐led nature of the programs, where students were not responsible for organizing course logistics or travel arrangements. The relatively low frequency of responses related to *c* *ommunication* (9.5%) is more surprising, given that verbal and written communication were central components of all three courses. However, it is quite possible that students did not perceive these skills as unique to the study abroad context, since they are commonly practiced in traditional on‐campus courses.

## 7 Discussion

This study contributes to the growing body of research demonstrating how short‐term, faculty‐led study abroad experiences serve as powerful contexts for leadership development. By mapping student outcomes to Seemiller's (2014) SLCs framework, the findings illustrated how intentional program design positively impact areas such as personal behavior, self‐awareness and development, civic responsibility, and learning and reasoning. These are qualities that contribute to strong future leadership as well as career readiness.

For leadership educators, this research underscores the importance of designing short‐term study abroad courses with explicit links to leadership competencies, so students gain much more than academic credit and a fun travel experience. When designing assignments for study abroad courses, faculty can use the SLC framework to develop reflection prompts, pre‐ and post‐program activities, and assessment strategies that foster LSE, intercultural competence, and critical thinking.

For higher education institutions, this study highlights the potential for short‐term study abroad to enhance both leadership education and career readiness. As long‐term travel may not be financially feasible for many students, short‐term programs can provide accessible, high‐impact learning opportunities. Integrating study abroad into leadership curricula can help colleges and universities demonstrate student learning outcomes connected to accreditation and employability frameworks such as NACE (2026) career readiness competencies.

Future research could include in‐depth interviews and focus groups with current students and graduates to gather more robust information about their growth in college and beyond as a result of their international study experience. Researchers could also analyze written documents such as pre‐ and post‐program reflections and academic essays. Generalizability of findings is limited, as only one university was studied; however, future research could refine the Qualtrics survey and administer it on a broader scale, sampling students at multiple universities with similar types of leadership study abroad programs.

## 8 Conclusion

This research is a starting point for leadership educators to utilize Seemiller's (2014) SLCs as a helpful framework for building effective short‐term study abroad programs. *The Student Leadership Competencies Guidebook* (Seemiller 2014) provides suggestions for how each competency can be incorporated into activities and initiatives. Faculty should intentionally build these competencies into their study abroad courses.

Colleges and universities with leadership development programs may begin to see the value of including study abroad programs as part of their offerings, and academic programs in leadership studies will hopefully see that courses like these are highly valuable for students. Practitioners can look beyond the significant academic learning that can take place in unique contexts throughout the world to see that these courses also provide rich opportunities for leadership development.
